# lncRNA DLGAP1-AS2 Knockdown Inhibits Hepatocellular Carcinoma Cell Migration and Invasion by Regulating miR-154-5p Methylation

**DOI:** 10.1155/2020/6575724

**Published:** 2020-10-09

**Authors:** Kai Chen, Zhuqing Zhang, Aijun Yu, Jian Li, Jinlong Liu, Xuejun Zhang

**Affiliations:** ^1^The First Department of General Surgery, Affiliated Hospital of Chengde Medical University, Chengde City, Hebei Province, 067000, China; ^2^Department of Clinical Laboratory, Affiliated Hospital of Chengde Medical University, Chengde City, Hebei Province, 067000, China

## Abstract

**Objective:**

DLGAP1-AS2 has been characterized as an oncogenic lncRNA in glioma. Our preliminary microarray analysis revealed the altered expression of DLGAP1-AS2 in hepatocellular carcinoma (HCC), but the role of DLGAP1-AS2 in HCC remains unknown.

**Method:**

Expression of DLGAP1-AS2 and miR-154-5p in paired HCC and nontumor tissues from 62 HCC patients was determined by RT-qPCR. The 62 HCC patients were followed up for 5 years to analyze the prognostic value of DLGAP1-AS2 for HCC. DLGAP1-AS2 knockdown and miR-154-5p overexpression was achieved in HCC cells to study the relationship between them. Methylation of miR-154-5p was analyzed by methylation-specific PCR. Cell proliferation was analyzed by CCK-8 assay.

**Results:**

DLGAP1-AS2 was upregulated in HCC and predicted poor survival. miR-154-5p was downregulated in HCC and inversely correlated with DLGAP1-AS2. In HCC cells, DLGAP1-AS2 knockdown resulted in the upregulation of miR-154-5p expression and decreased methylation of miR-154-5p gene. Transwell assay showed that DLGAP1-AS2 knockdown and miR-154-5p overexpression inhibited cell invasion and migration, and the combination of LGAP1-AS2 knockdown and miR-154-5p overexpression showed stronger effects.

**Conclusion:**

DLGAP1-AS2 knockdown may inhibit HCC cell migration and invasion by regulating miR-154-5p methylation.

## 1. Introduction

Liver cancer is a common malignancy for both incidence and mortality [1]. It is reported that liver cancer in 2018 caused a total of 781,631 deaths, which were the 8.2% of all cancer deaths and affected a total of 841,080, accounting for 4.7% of all new cancer cases [2]. Hepatocellular carcinoma (HCC) is the major subtype of liver cancer [3]. It is reported that the median survival time of HCC patients is only 6 to 20 months, and only less than 10% of HCC patients can survive for more than 5 years after the initial diagnosis [4]. Therefore, novel treatments are needed to improve the survival of HCC patients. Hepatitis B or Hepatitis C infections, alcohol consumptions, and obesity are the main risk factors for HCC [5–7]. However, molecular pathogenesis of HCC is still unclear. Therefore, the development of novel anti-HCC approaches is limited.

It has been well established that the development and progression of HCC require the involvement of molecular alterations [8, 9]. Functional characterization of these molecular factors facilitates the development of novel therapies, such as targeted therapy that can be applied to suppress cancers by regulation cancer-related gene expression network [10]. Despite of the lacking of protein-coding capacity, noncoding RNAs (ncRNAs), such as miRNAs and lncRNAs, can regulate cancer progression by affecting gene expression [11, 12]. Therefore, ncRNAs are promising targets for cancer treatment, and function analysis of the roles of ncRNAs is required. In a recent study, DPLGAP1-AS2 has been characterized as an oncogenic lncRNA in glioma [13], while its role in other cancers is unknown. Our deep sequencing analysis revealed the alteration of DLGAP1-AS2 in HCC and its inverse correlation with miR-154-5p (data not shown), which is also a critical player in cancer biology [14]. For instance, miR-154-5p is underexpressed in glioblastoma and its overexpression targets PIWIL1 to inhibit cell proliferation and metastasis [14]. However, the role of miR-154-5p in HCC is unknown. This study was therefore carried out to analyze the interactions between DLGAP1-AS2 and miR-154-5p in HCC.

## 2. Materials and Methods

### 2.1. HCC Patients

At Affiliated Hospital of Chengde Medical University, hospital, we enrolled 62 patients (36 males and 26 females) from the 122 HCC patients admitted to the aforementioned hospital from May 2012 to January 2015. Age of the patients ranged from 42 years to 68 years (54.8 ± 5.8 years). Ethics Committee of the aforementioned hospital approved this study. All these HCC patients were confirmed by histopathological exam. Recurrent HCC patients (*n* = 30) were excluded, and all the 62 HCC patients were newly diagnosed cases. It is known that gene expression may be affected by therapies and other clinical disorders; this study excluded patients complicated with other clinical disorders (*n* = 15) and the ones with initiated therapy (12). The ones who died of causes unrelated to HCC during follow-up (will be mentioned below, *n* = 3) were also excluded. Finally, only 62 patients were included in analysis. Based on medical record, the 62 patients included 27 cases of HBV positive, 26 cases of HCV positive, and 9 cases of negative for both. All patients signed informed consent.

### 2.2. Treatment and Follow-Up

The 62 patients were classified into AJCC stages I and II (*n* = 28), and III and IV (*n* = 34). Treatment approaches were determined based on American Joint Committee on Cancer (AJCC) stages and the health conditions of patients. The 62 patients were followed up for 5 years through telephone in a monthly manner. Patients' survival conditions were recorded.

### 2.3. HCC Tissues and Cells

Fine needle aspiration was performed to obtain HCC and adjacent (within 3 cm around tumors) paired nontumor liver tissues from the 62 HCC patients. After histopathological confirmation, all tissue samples were immediately subjected to RNA isolation. HCC cell line SNU-398 (ATCC, USA) was included. RPMI 1640 (90%) was mixed with FBS (10%) to serve as culture medium for SNU-398 cells. In a 5% CO_2_ and 95% humidity, incubator cells were cultivated at 37°C.

### 2.4. Transient Transfections

DLGAP1-AS2 siRNA and negative control (NC) siRNA, as well as NC miRNA and mimic of miR-154-5p, were all purchased from Invitrogen (Shanghai, China). Through Lipofectamine 2000- (Invitrogen-) mediated transient transfections, SNU-398 cells were transfected with 40 nM siRNA or miRNA. To perform NC experiments, cells were transfected with either NC siRNA or NC miRNA. Incubation with transfection mixture was performed for 6 h, followed by cell culture in fresh medium for further 48 h. To perform control (C) experiments, untransfected cells were cultivated in fresh medium for 54 h.

### 2.5. RNA Isolation and Process

SNU-398 cells and paired tissue samples were subjected to RNA isolations using RNAzol (Invitrogen). Incubation with DNase I (Invitrogen) was performed for 100 min at 37°C to achieve complete genomic DNA removal. Ratios of OD260/280 were determined to check RNA purity.

### 2.6. RT-qPCR

Reverse transcriptions (RTs) were performed using RNA samples with a ratio of OD260/280 with the SSRT IV system (Invitrogen) to prepare cDNA samples. All cDNAs were subjected to qPCRs using GeneRead qPCR SYBR Green Master Mix (QIAGEN) to determine the expression of DLGAP1-AS2. Internal control of DLGAP1-AS2 was 18S rRNA. To determine the expression of miR-154-5p, mature miRNAs were added with poly(A), followed by miRNA RTs and qPCRs. U6 was used as internal control of miR-154-5p. Each experiment was performed in three technical replicates, and the 2^-*ΔΔ*Ct^ method was used for Ct value normalizations.

### 2.7. Methylation-Specific PCR (MSP)

Genomic DNAs were extracted from SNU-398 cells with DLGAP1-AS2 siRNA silencing. Genomic DNA Extraction Kit (ab156900, Abcam) was used. DNA samples were converted using the EZ DNA Methylation Lighting Kit (ZYMO research), followed by PCRs to determine the methylation of miR-154-5p.

### 2.8. Transwell Assays

Invasion and migration of SNU-398 cells were determined using Transwell filters (8 *μ*m, Dojindo). To perform invasion assay, Matrigel (Dojindo) was used to coat membranes for 12 h at 37°C to mimic *in vivo* invasion conditions. In contrast, uncoated membranes were used in migration assay. Cells were transferred to upper Transwell chamber with 5000 cells in 0.1 ml serum-free medium and the lower chamber were filled with medium containing 20% FBS to induce cell migration and invasion. At 37°C, cells were cultivated for 12 h, and the upper surface of membranes was cleaned with a cotton swab. Crystal violet (0.1%) was used to stain the lower surface of membranes for 20 min. A light microscope was used to observe cells.

### 2.9. Statistical Analysis

Gene expression in paired tissues from the same patient was expressed as average values of 3 technical replicates and compared by paired *t* test. Data of multiple transfection groups were expressed as mean ± SD values of 3 biological replicates and compared by ANOVA Tukey's test. Correlations were analyzed by linear regression. With the median level of DLGAP1-AS2 expression in HCC tissues as a cutoff value, the 62 patients were divided into high and low DLGAP1-AS2 level groups (*n* = 31). Survival curves were plotted and compared by the log-rank test. *p* < 0.05 was deemed statistically significant.

## 3. Results

### 3.1. A High Level of DLGAP1-AS2 in HCC Predicted Poor Survival

**Figure 1:**
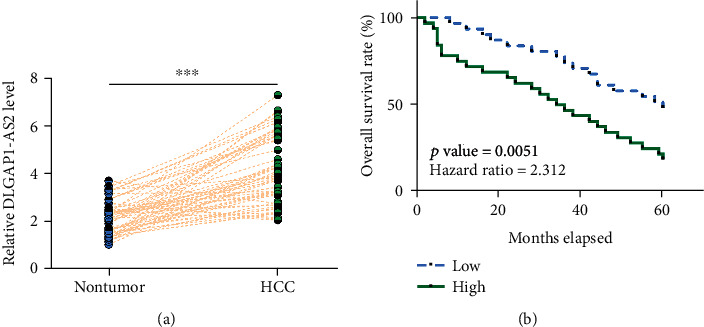
A high level of DLGAP1-AS2 in HCC predicted poor survival. Expression of DLGAP1-AS2 in paired HCC and nontumor tissues was determined by RT-qPCR. Gene expression levels in paired tissues were expressed as average values of 3 technical replicates (a). ∗∗∗*p* < 0.001. With median level of DLGAP1-AS2 expression in HCC tissues as a cutoff value, the 62 patients were divided into high and low DLGAP1-AS2 level groups (*n* = 31). Survival curves were plotted and compared by the log-rank test (b).

Expression of DLGAP1-AS2 in paired HCC and nontumor tissues was determined by RT-qPCR. Compared with nontumor tissues, HCC tissues exhibited significantly higher DLGAP1-AS2 expression (Figure 1(a); *p* < 0.001). Survival curve analysis showed that patients in the high DLGAP1-AS2 level group exhibited significantly higher mortality rate in comparison to patients in the low DLGAP1-AS2 level group (Figure 1(b)). Therefore, DLGAP1-AS2 is overexpressed in HCC, and its high expression levels in HCC predicted poor survival. It is worth noting that no significant differences in the expression of DLGAP1-AS2 in HCC tissues was found among HBV-positive, HCV-positive, and both-negative groups (Figure S1(A)).

### 3.2. miR-154-5p Is Downregulated in HCC and Was Inversely Correlated with DLGAP1-AS2

**Figure 2:**
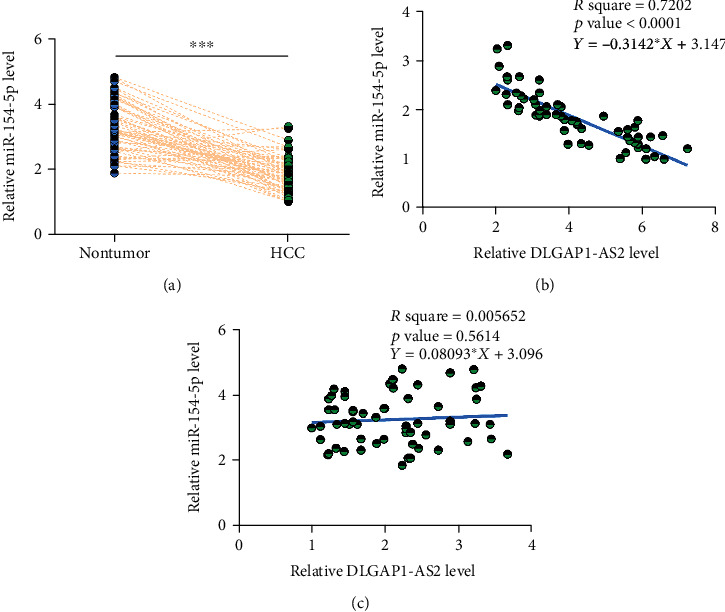
miR-154-5p is downregulated in HCC and was inversely correlated with DLGAP1-AS2. Expression of miR-154-5p in paired HCC and nontumor tissues was determined by RT-qPCR. Gene expression levels in paired tissues were expressed as average values of 3 technical replicates (a). ∗∗∗*p* < 0.001. Linear regression was performed to analyze the correlations between miR-154-5p and DLGAP1-AS2 across HCC tissues (b), but not across nontumor tissues (c).

Expression of miR-154-5p in paired HCC and nontumor tissues was determined by RT-qPCR. Compared with nontumor tissues, HCC tissues exhibited significantly lower levels of miR-154-5p expression (Figure 2(a); *p* < 0.001). Correlation analysis showed that miR-154-5p was inversely correlated with DLGAP1-AS2 across HCC tissues (Figure 2(b)), but not across nontumor tissues (Figure 2(c)). Therefore, DLGAP1-AS2 and miR-154-5p may interact with each other in HCC. It is noteworthy that no significant differences in the expression of miR-154-5p in HCC tissues were found among HBV-positive, HCV-positive, and both-negative groups (Figure S1(B)).

### 3.3. DLGAP1-AS2 Knockdown Upregulated miR-154-5p through Methylation

**Figure 3:**
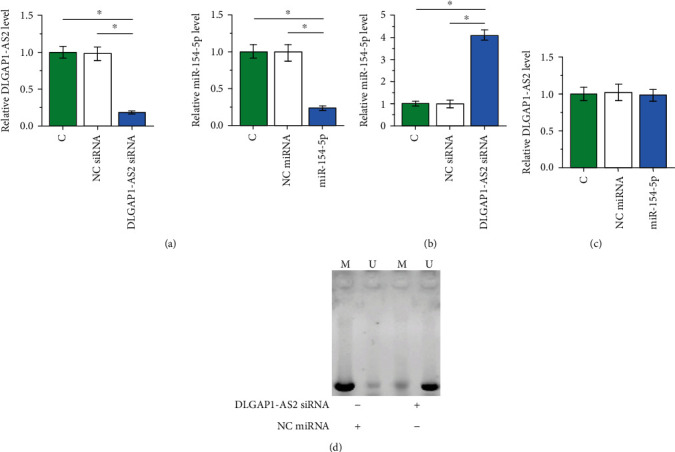
DLGAP1-AS2 knockdown upregulated miR-154-5p through methylation. To explore the interaction between DLGAP1-AS2 and miR-154-5p, SNU-398 cells were transfected with DLGAP1-AS2 siRNA or miR-154-5p mimic, followed by the confirmation of transfections by RT-qPCR (a). The effects of DLGAP1-AS2 siRNA silencing on miR-154-5p (b) or the effects of miR-154-5p overexpression on DLGAP1-AS2 (c) were also analyzed by RT-qPCR. MSP was performed to analyze the methylation of miR-154-5p in cells with DLGAP1-AS2 siRNA or NC siRNA transfection (d). Data of multiple transfection groups were expressed as mean ± SD values of 3 biological replicates. M: methylation; U: unmethylation. ∗*p* < 0.05.

To explore the interaction between DLGAP1-AS2 and miR-154-5p, SNU-398 cells were transfected with DLGAP1-AS2 siRNA or miR-154-5p mimic, followed by the confirmation of transfections by RT-qPCR (Figure 3(a); *p* < 0.05). It was observed that cells with DLGAP1-AS2 siRNA showed significantly higher miR-154-5p expression (Figure 3(b); *p* < 0.05). In contrast, cells with miR-154-5p overexpression showed no significant alterations in DLGAP1-AS2 expression (Figure 3(c)). MSP was performed to analyze the methylation of miR-154-5p. Compared with cells transfected with NC siRNA, cells transfected with DLGAP1-AS2 siRNA silencing showed decreased methylation of miR-154-5p gene (Figure 3(d)).

### 3.4. DLGAP1-AS2 siRNA Silencing and miR-154-5p Overexpression Inhibited Cell Invasion and Migration

**Figure 4:**
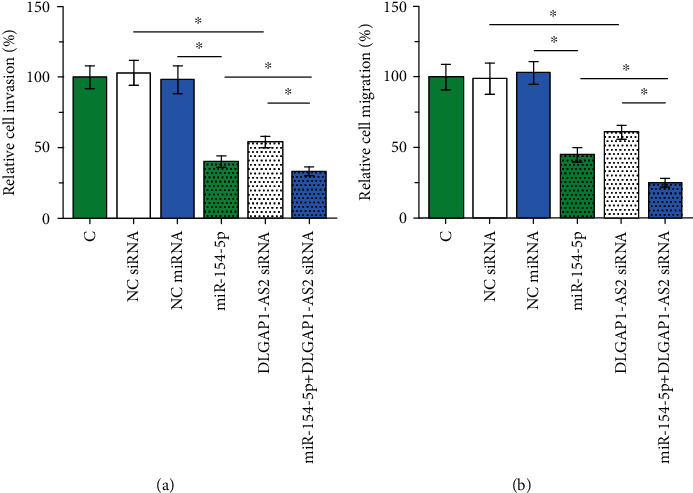
DLGAP1-AS2 siRNA silencing and miR-154-5p overexpression inhibited cell invasion and migration. Transwell assays were performed to analyze the effects of DLGAP1-AS2 siRNA silencing and miR-154-5p overexpression on the cell invasion (a) and migration (b) of SNU-398 cells. Data of multiple transfection groups were expressed as mean ± SD values of 3 biological replicates. ∗*p* < 0.05.

Transwell assay showed that DLGAP1-AS2 knockdown and miR-154-5p overexpression inhibited cell invasion (Figure 4(a)) and migration (Figure 4(b)), and the combination of LGAP1-AS2 knockdown and miR-154-5p overexpression showed stronger effects (*p* < 0.05).

## 4. Discussion

This study analyzed the roles of DLGAP1-AS2 and miR-154-5p in HCC and explored the interaction between them. We found that DLGAP1-AS2 was upregulated in HCC and could regulate the methylation of miR-154-5p gene to regulate the invasion and migration of HCC cells.

The functionality of DLGAP1-AS2 has only been characterized in glioma [13]. DLGAP1-AS2 is overexpressed in glioma and may target Yes Associated Protein 1 to suppress cancer cell apoptosis but promote cell proliferation and migration [13]. In this study, we observed the upregulation of DLGAP1-AS2 in HCC and decreased invasion and migration of HCC cells after DLGAP1-AS2 knockdown. Therefore, DLGAP1-AS2 is likely an oncogenic lncRNA in HCC, and DLGAP1-AS2 knockdown may be a potential target for the treatment of HCC.

Most HCC patients were diagnosed at late stages, leading to poor prognosis [15]. Effective tumor markers for the early diagnosis of HCC remain lacking. As the consequence, early diagnosis of HCC is unlikely to be improved in the near future [16]. In this study, we found that high DLGAP1-AS2 expression in HCC tissues were correlated with the poor survival of HCC patients. Therefore, DLGAP1-AS2 may be used as a tumor marker to assist the prognosis of HCC patients, thereby guiding the determination of treatments and improving patients' survival.

miR-154-5p plays different roles in different types of cancers [14, 17]. miR-154-5p is downregulated in glioma and targets PIWIL1 to suppress tumor growth and metastasis [14]. In contrast, miR-154-5p is overexpressed in renal cell carcinoma and promotes cancer cell migration and proliferation [17]. In this study, we first reported the upregulation of miR-154-5p in HCC and inhibitory effects on cancer cell invasion and migration. Therefore, miR-154-5p may serve as a tumor suppressor in HCC. The upstream regulator of miR-154-5p in cancer biology has not been reported by previous studies. In this study, we showed that DLGAP1-AS2 may regulate the methylation of miR-154-5p to affect its expression, while the mechanism is unknown. Interestingly, miR-154-5p and DLGAP1-AS2 were only correlated across HCC tissues, but not across nontumor tissues. Therefore, certain pathological factors may mediate the interaction between them in HCC.

HBV and HCV infections are the major cause of HCC [5]. However, in this study, we found that the expression levels of DLGAP1-AS2 and miR-154-5p were not significantly different among HBV-positive, HCV-positive, and both-negative group. Therefore, DLGAP1-AS2 and miR-154-5p may participate in HCC through HBV-/HCV-independent pathways.

This study is limited by the small sample size. In addition, the *in vivo* interaction between DLGAP1-AS2 and miR-154-5p in HCC is unknown. Future studies with bigger sample size and *in vivo* animal model studies are needed to further confirm the interaction between them.

In conclusion, upregulation of DLGAP1-AS2 in HCC predicts the poor survival of HCC patients. In addition, DLGAP1-AS2 may regulate the methylation of miR-154-5p gene to regulate the invasion and migration of HCC cells.

## Data Availability

Readers can email the authors (KaiChen1965@163.com) for accessing the data used in the manuscript.

## Supplementary Materials

Supplementary Materials Fig S1: comparison of the expression levels of DLGAP1-AS2 (A) and miR-154-5p (B) in HCC tissues among HBV-positive, HCV–positive, and both-negative groups.
