# Supplementary figures and images for: lncRNA DLGAP1-AS2 Knockdown Inhibits Hepatocellular Carcinoma Cell Migration and Invasion by Regulating miR-154-5p Methylation
Source: Biomed Res Int. 2020 Oct 9;2020:6575724. doi: 10.1155/2020/6575724 (PMC7641292; doi:10.1155/2020/6575724)

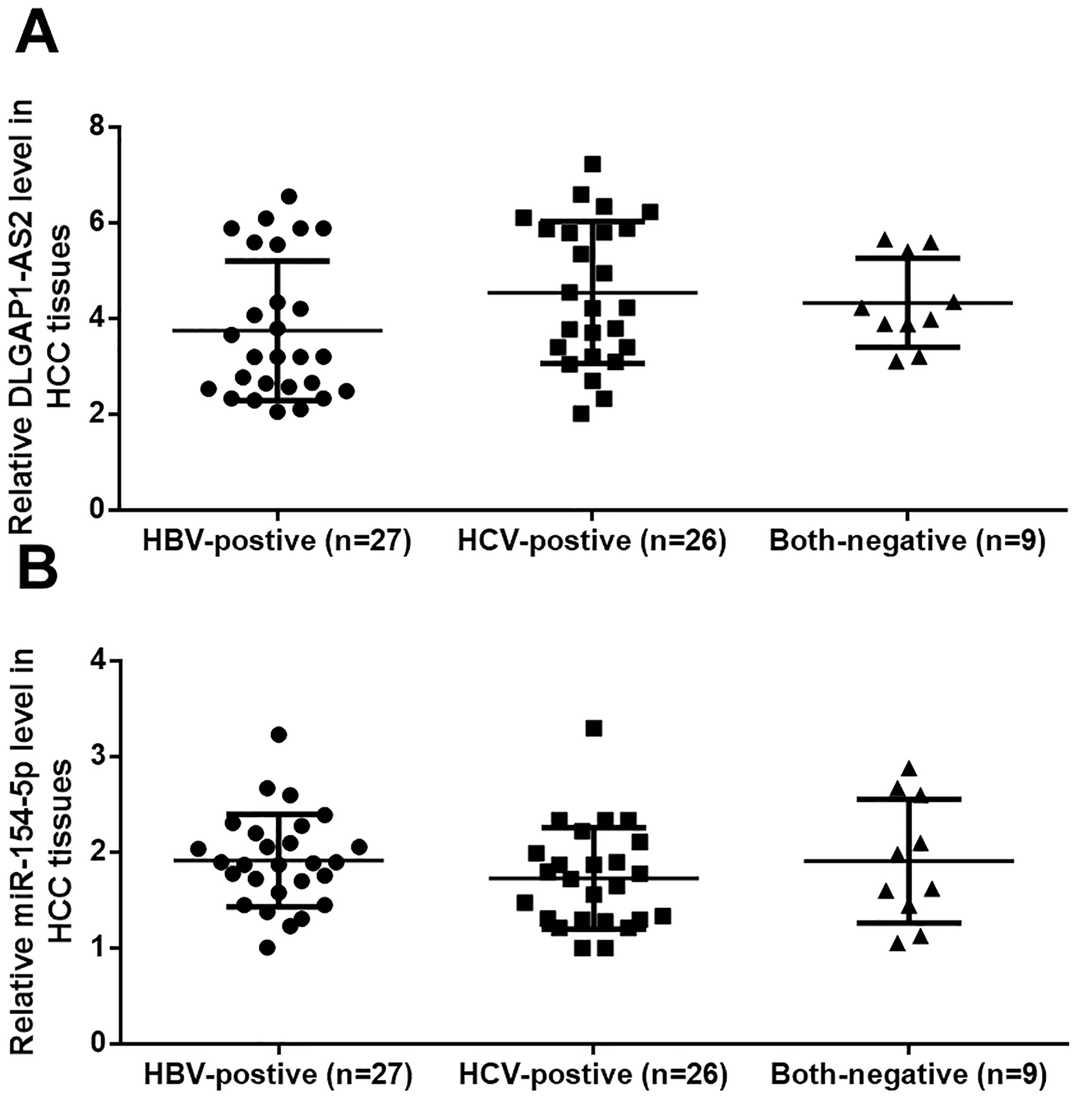

Supplement: Supplementary Materials — Fig S1: comparison of the expression levels of DLGAP1-AS2 (A) and miR-154-5p (B) in HCC tissues among HBV-positive, HCV–positive, and both-negative groups. [file 6575724.f1.tif]
